# PRDM Family Proteins in Immune Regulation: Epigenetic Control and Implications in Immune-Related Diseases

**DOI:** 10.3390/biology15100801

**Published:** 2026-05-18

**Authors:** Shiqi Deng, Hui Li, Chengyan Guo, Jun Zou, Lingli Zhang

**Affiliations:** 1 College of Athletics Performance, Shanghai University of Sport, Shanghai 200438, China; 2 Faculty of Pharmaceutical Sciences, Shenzhen University of Advanced Technology, Shenzhen 518107, China; 3 Center for AI-Driven Medical Research, Shenzhen Institutes of Advanced Technology, Chinese Academy of Sciences, Shenzhen 518055, China; 4 School of Exercise and Health, Shanghai University of Sport, Shanghai 200438, China

**Keywords:** PRDM family, epigenetic regulation, immune regulation, hematopoiesis, inflammatory signaling, immunometabolism

## Abstract

PRDM family proteins are a group of epigenetic regulators characterized by a conserved PR domain and multiple zinc-finger motifs. To date, eighteen PRDM family members have been identified in both mice and humans. Increasing evidence indicates that PRDM members play critical roles in the regulation of immune responses and immune-related diseases. They are involved in key processes such as hematopoiesis, immune cell differentiation, inflammatory signaling, and immunometabolic regulation. Dysregulation of PRDM proteins has been associated with a variety of pathological conditions, including hematological malignancies, autoimmune and inflammatory diseases, and metabolic disorders. Notably, functional studies of PRDM family members remain highly uneven, with a few proteins extensively characterized while many others are still poorly understood. This review summarizes recent advances in the structural features and biological functions of PRDM proteins, with a particular focus on their roles in immune regulation and endocrine–metabolic homeostasis. Furthermore, we discuss their involvement in disease pathogenesis and highlight their potential as therapeutic targets. Overall, this work provides a systematic overview of PRDM family functions and offers insights for future research in immune-mediated and metabolic diseases.

## Simple Summary

The immune system plays a central role in protecting the body from infections and maintaining overall health. When immune regulation becomes disrupted, it can contribute to the development of many diseases, including autoimmune disorders, chronic inflammation, metabolic diseases, and blood cancers. PRDM family proteins are a group of regulators that help control how genes are turned on or off in immune cells and other tissues. Increasing evidence shows that these proteins are involved in immune cell development, inflammatory responses, and metabolic balance. However, knowledge about many PRDM family members remains limited. In this review, we summarize current research on the structure and biological functions of PRDM proteins, with a focus on their roles in immune regulation and disease development. We also discuss their potential value as therapeutic targets for immune-related and metabolic diseases. This review provides an overview of how PRDM proteins influence immune health and may support the development of new strategies for disease prevention and treatment.

## 1. Introduction

The immune system is an interactive network composed of lymphoid organs, immune cells, humoral factors, and cytokines, which collectively coordinate host defense and immune surveillance [1,2]. Its precise regulation depends on highly coordinated transcriptional and epigenetic programs. These regulatory networks integrate external stimuli with intracellular signaling cues and thereby control the spatiotemporal expression of immune-related genes [3,4]. Through this process, transcriptional and epigenetic regulators orchestrate immune cell lineage commitment [5], regulate effector functions, and maintain immune homeostasis [6].

Transcriptional regulatory proteins are a group of proteins that control gene expression by directly or indirectly regulating transcription [7]. Among them, PRDM proteins represent an important group of transcriptional regulators. Members of this family share highly conserved structural features, including an N-terminal PR (PRDI-BF1 and RIZ homology) domain and a variable number of classical C2H2 zinc-finger motifs arranged toward the C-terminus [8]. The PR domain at the N-terminus is considered to possess potential histone methyltransferase activity, whereas the C-terminal zinc fingers are thought to mediate protein–protein, protein-RNA, or protein-DNA interactions [9,10]. Most PRDM genes generate two major transcriptional isoforms. One isoform lacks the PR domain (PR-negative), whereas the other retains the PR domain (PR-positive) [10]. These isoforms are usually generated through alternative promoter usage or alternative splicing. They are regulated by distinct mechanisms and often exert different, or even opposing, functions in diverse physiological and pathological processes [11]. Previous studies have mainly focused on their roles in cancer, where loss, mutation, epigenetic silencing, or aberrant expression of PRDM isoforms has been closely associated with tumor initiation and progression [12,13]. Notably, similar isoform-specific regulatory mechanisms have increasingly attracted attention in the immune system. PRDM proteins regulate transcriptional programs of immune-related genes and thereby participate in immune cell differentiation, functional specialization, and the fine regulation of immune responses [14].

Although members of the PRDM family have been relatively well studied in areas such as nervous system development, tumorigenesis, and metabolic regulation [9,10,15], their roles in the immune system have not yet been comprehensively summarized. In recent years, increasing evidence has shown that PRDM proteins participate in multiple key aspects of immune regulation. These include immune cell lineage commitment, functional maturation, regulation of inflammatory responses, and the development of immune-related diseases [16,17]. However, most existing studies have focused on individual PRDM members or specific immune contexts. As a result, the overall biological significance of the PRDM family in immune regulation remains insufficiently understood.

Therefore, this review aims to systematically summarize recent advances in understanding the roles of the PRDM family in the immune system. In particular, it focuses on the molecular mechanisms through which PRDM proteins participate in immune regulation, including their involvement in transcriptional and epigenetic regulatory networks. In addition, this review discusses the potential roles of PRDM proteins in various immune-related diseases. By integrating current findings, this work seeks to provide a conceptual framework for understanding PRDM-mediated immune regulation and to offer a theoretical basis for future mechanistic studies and the development of immune intervention strategies.

## 2. Epigenetic Regulation by the PRDM Family in the Immune System

### 2.1. Structural Features of the PRDM Family

The PRDM (PRDI-BF1 and RIZ homology domain-containing) protein family represents a conserved group of transcriptional and epigenetic regulators. To date, multiple members have been identified in mammals, including PRDM1–PRDM16 as well as FOG1 and FOG2 [8]. Structurally, typical PRDM proteins contain an N-terminal PR (PRDI-BF1 and RIZ homology) or SET-like domain and a variable number of classical C2H2 zinc-finger motifs arranged toward the C-terminus. The PR domain is highly similar to the SET domain in both sequence and spatial conformation. As a result, some PRDM proteins possess potential histone methyltransferase activity. In addition, the PR domain can serve as a platform to recruit histone modification-related complexes, thereby contributing to the regulation of chromatin states [8]. The C-terminal C2H2 zinc-finger motifs are primarily responsible for DNA sequence recognition and protein–protein interactions, thereby enabling PRDM proteins to precisely target specific gene regulatory regions [11]. Importantly, the functional properties of PRDM proteins are not uniform: while some members possess intrinsic or putative histone methyltransferase activity, others primarily function as transcriptional regulators by recruiting chromatin-modifying complexes [11].

Different PRDM members exhibit distinct patterns of tissue distribution, target gene selection, and biological functions. They have been reported to participate in various physiological and pathological processes, including nervous system development, tumorigenesis, metabolic regulation, and immune responses [18,19,20,21,22]. To provide a clearer comparative framework, the structural features, and immune-related functions of PRDM family members are summarized in Table 1.

### 2.2. PRDM-m2.2 PRDM-Mediated Immune-Related Epigenetic Regulation

The development and functional regulation of the immune system depend on the precise modulation of epigenetic mechanisms, including DNA methylation and histone modifications [46]. This higher-level regulatory framework plays a critical role in maintaining immune homeostasis and enabling adaptive responses to environmental changes [47]. Proteins of the PRDM family play important roles in immune-related epigenetic regulatory networks. For instance, the PR/SET domains of PRDM3 and PRDM16 can interact with the core component RBBP4 of the NuRD complex, thereby recruiting the remodeling complex to specific genomic loci and contributing to local chromatin structural remodeling [31].

From a functional perspective, PRDM proteins not only act as histone-modifying enzymes but also function as epigenetic regulatory platforms that recruit and integrate co-regulatory complexes [48]. In addition, in Th1 and Th17 cells, PRDM1/Blimp-1 directly binds to the promoters of immune cytokine genes, such as IL-21, where it reduces chromatin accessibility and promotes the enrichment of repressive histone marks, including H3K9me3 and H3K27me3. These observations further support the notion that PRDM proteins regulate immune gene expression by modulating local chromatin architecture [49].

It is worth noting that PRDM-mediated epigenetic regulation exhibits strong context dependency, and its transcriptional activating or repressive effects are determined by the cellular microenvironment, upstream signaling inputs, and the composition of co-regulatory complexes [11]. The dynamic and reversible nature of this regulation enables PRDM proteins to fine-tune immune gene expression programs, thereby contributing to immune homeostasis and influencing immune-related pathologies.

## 3. Roles of the PRDM Family in Immune Cell Development and Differentiation

**Figure 1:**
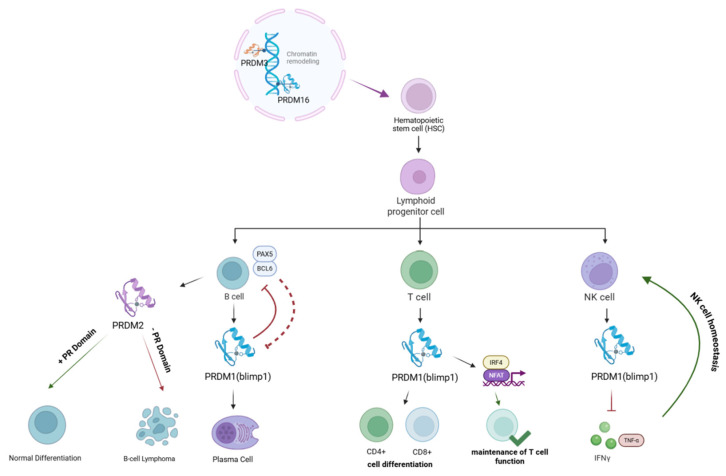
PRDM family members regulate lymphocyte differentiation and immune cell function. PRDM3 and PRDM16 regulate chromatin remodeling during hematopoietic stem cell differentiation and lymphoid lineage commitment. PRDM1 (Blimp-1) promotes plasma cell differentiation in B cells and regulates effector programs in T cells through interactions with transcription factors such as IRF4 and NFAT. PRDM2 (RIZ1) is implicated in B-cell differentiation and lymphoma development, while PRDM1 also maintains NK cell homeostasis by limiting inflammatory cytokine production.

The immune system comprises innate and adaptive components that coordinate host defense through tightly regulated transcriptional and epigenetic programs. Within this regulatory framework, PRDM family proteins have emerged as key modulators of immune cell lineage commitment and functional differentiation. Among these regulators, members of the PRDM family (Figure 1) play important roles in hematopoietic stem cell fate determination and the regulation of lymphocyte differentiation programs [10,16,42].

### 3.1. PRDM Family in Adaptive Immune Cell Development

#### 3.1.1. PRDM Proteins in B Cell Development and Differentiation

B cells play a key role in humoral immune responses, and their development and differentiation are tightly regulated by multilayered molecular mechanisms [50]. The PRDM family comprises transcriptional and epigenetic regulators that play important roles in B cell fate determination and terminal differentiation [16]. The gene PRDM1, which encodes human BLIMP1, is located on chromosome 6q21, a locus that is frequently deleted in lymphomas. PRDM1 can silence its target genes through multiple mechanisms in a context-dependent manner. It is expressed in both B cells and T cells, where it performs important regulatory functions. In B cells, PRDM1 acts as a master regulator of plasma cell differentiation. Its expression is repressed by BCL6, whereas PRDM1 in turn suppresses the expression of BCL6 and PAX5 [24], thereby promoting the establishment of the secretory program characteristic of mature B cells. Another study used a mouse monoclonal antibody specific for PRDM1 to analyze the distribution of PRDM1 in normal human lymphoid tissues and in lymphomas corresponding to different stages of B-cell differentiation. PRDM1 expression was detected in germinal center blasts, which co-expressed Pax5, CD19, CD20, and CD10 but lacked expression of BCL6 and MTA-3, suggesting that PRDM1 expression is associated with the transition of germinal center B cells toward plasma cell differentiation [51].

RIZ1 (PRDM2) has been reported to participate in B-cell differentiation and in the development and progression of B-cell lymphomas [29]. Notably, PRDM2 is expressed as two isoforms that differ in the presence or absence of the PR domain. The PR domain-containing isoform exhibits tumor-suppressive activity, whereas the isoform lacking the PR domain displays oncogenic properties and is frequently overexpressed in tumor cells [30]. This “yin–yang” regulatory pattern enables the two isoforms to exert opposing effects in cell fate determination and tumor development. Dysregulated expression of PRDM2 and its isoforms has been observed in multiple tumor types, including lymphoid malignancies [52]. Consistent with these observations, PRDM2/RIZ1 has been suggested to interact with B cell differentiation-related signaling networks to regulate chromatin states and the expression of key genes during lymphocyte development [28,29], indicating a potential role in B cell differentiation and in the pathogenesis of B-cell lymphomas.

#### 3.1.2. PRDM Proteins in T Cell Development and Functional Differentiation

T cells represent another major component of the adaptive immune system, and their development and functional differentiation also depend on precisely coordinated transcriptional regulatory networks [53,54]. In T cells, PRDM11 is an important factor that promotes the differentiation of most terminal effector cells in both CD4^+^ and CD8^+^ T cell lineages [24]. Recent studies have shown that PRDM1 not only acts as a classical transcriptional repressor regulating immune cell differentiation but also contributes to adaptive immune responses by remodeling chromatin accessibility and epigenetic states [49]. In T cells, PRDM1 cooperates with transcription factors such as IRF4 to integrate signals derived from persistent antigen stimulation [25]. Through this process, it regulates the balance between effector-related gene expression and inhibitory programs, thereby contributing to the maintenance of effector T cell functions and the development of T cell exhaustion [55].

In addition to PRDM1, which is the most extensively studied regulator of adaptive immune responses, emerging evidence suggests that other PRDM family members may also contribute to lymphoid development, particularly at early stages, based on their conserved structural features and regulatory functions observed in other cellular lineages. These proteins are proposed to influence T cell fate decisions through epigenetic regulation and interactions with core transcription factor networks [43]. For instance, PRDM14 has been shown to regulate lymphoid progenitor expansion and is implicated in the initiation of lymphoid malignancies, including T-cell acute lymphoblastic leukemia, indicating a role in early T-lineage specification [39,40]. Moreover, PRDM16 plays a critical role in the maintenance of hematopoietic stem cells and early progenitor populations, which give rise to T lymphocytes [42]. Although its direct role in mature T-cell function remains unclear, these findings suggest that PRDM16 may indirectly influence T-cell development through upstream regulation of hematopoietic differentiation.

Overall, while PRDM1 remains the central regulator in mature T-cell differentiation and function, other PRDM family members may contribute to adaptive immunity by modulating early lymphoid commitment and progenitor cell dynamics. However, their specific roles in mature T-cell subsets are still poorly defined and warrant further investigation.

### 3.2. PRDM Family in Innate Immune Cell Differentiation and Functional Programming

Innate immune cells constitute the first line of host defense against pathogens, and their differentiation and functional activities are tightly regulated by transcriptional and epigenetic mechanisms [56]. Representative innate immune cells include natural killer (NK) cells, dendritic cells (DCs), and macrophages, which recognize pathogen-associated patterns through pattern recognition receptors (PRRs), thereby rapidly initiating defensive responses such as cytotoxic activity and inflammatory signaling [57,58,59].

During this process, members of the PRDM family are broadly involved in the regulation of key gene programs. PRDM1 (Blimp-1), one of the earliest identified members of the PRDM family, functions as a negative regulator in NK cells. It suppresses excessive activation by directly inhibiting the production of inflammatory cytokines such as IFN-γ and TNF-α, while also contributing to terminal differentiation and the maintenance of cellular homeostasis [26]. Another multi-omics study further revealed the role of PRDM1 in NK cell terminal differentiation and homeostasis, highlighting the critical importance of Blimp-1 during the maturation stage of NK cells, with loss of its activity being associated with increased malignancy [14].

In addition, factors such as PRDM3 and PRDM16 may influence the formation of innate immune lineages by regulating the fate and epigenetic state of hematopoietic stem cells [32], highlighting the importance of epigenetic networks during the early stages of innate immune cell differentiation. Although the specific roles of PRDM proteins in other innate immune cell types, such as macrophages and dendritic cells, remain insufficiently characterized, emerging evidence suggests that this transcriptional and epigenetic regulatory family contributes to the regulation of immune lineage development.

## 4. PRDM Family in Immune-Related Diseases

Given their central roles in immune cell development and functional differentiation, PRDM proteins are essential for maintaining immune homeostasis. Consequently, dysregulation of these regulatory networks can disrupt immune balance and contribute to the development of immune-related diseases.

### 4.1. Hematologic Malignancies

**Figure 2:**
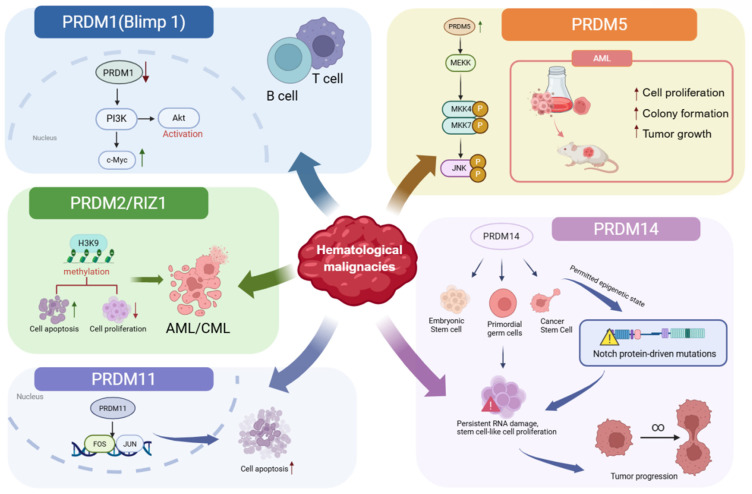
PRDM family members in hematological malignancies. Schematic overview of the distinct functions of PRDM family proteins in hematologic cancers. PRDM1 (Blimp-1) acts as a tumor suppressor in lymphoid malignancies and is associated with PI3K/AKT signaling dysregulation in B- and T-cell lymphomas. PRDM2/RIZ1 suppresses leukemogenesis through histone H3K9 methylation and regulation of apoptosis and proliferation in AML and CML. In contrast, PRDM5 promotes tumor growth in acute myeloid leukemia through activation of the JNK signaling pathway. PRDM11 functions as a tumor suppressor in diffuse large B-cell lymphoma by regulating oncogenic transcription factors such as FOS and JUN. PRDM14 contributes to leukemogenesis by promoting stem-like programs and facilitating oncogenic mutations such as NOTCH1.

Hematologic malignancies are a group of clonal cancers that arise from hematopoietic stem/progenitor cells or mature immune cells within the blood, bone marrow, and lymphatic system, and primarily include leukemia, lymphoma, and multiple myeloma [60]. These malignancies exhibit substantial heterogeneity in both clinical manifestations and cellular origins, and their initiation and progression are closely associated with the accumulation of genetic mutations and epigenetic dysregulation [61]. Members of the PRDM family play important oncogenic or tumor-suppressive roles in hematologic malignancies (Figure 2), and their aberrant expression or dysregulated activity can markedly influence tumor initiation, progression, and clinical prognosis.

PRDM1 (Blimp-1) functions as a tumor suppressor and plays an important pathological role in B-cell and T-cell lymphomas [62]. Loss of PRDM1 is frequently associated with aberrant activation of the PI3K/AKT signaling pathway and correlates with poor clinical outcomes. Moreover, the PRDM1β isoform has been linked to increased c-MYC expression, further suggesting that dysregulation of PRDM1 expression and isoform balance contributes to lymphoma progression and influences patient survival [18].

PRDM2, also known as RIZ (retinoblastoma protein-interacting zinc finger protein), has been closely implicated in hematologic malignancies. The RIZ gene, located on chromosome 1p36, has been identified as a tumor suppressor locus [63]. It encodes two isoforms, RIZ1 and RIZ2, generated through alternative promoter usage. RIZ1 contains the N-terminal PR domain, whereas RIZ2 lacks this domain. Altered expression of RIZ has been reported in multiple human cancers, and RIZ1 in particular is widely regarded as a candidate tumor suppressor [64]. Mechanistically, RIZ1 functions as a histone methyltransferase that catalyzes the methylation of histone H3 at lysine 9 (H3K9), a modification associated with transcriptional repression [65]. Consistent with its tumor-suppressive role, reduced RIZ1 expression has been observed in acute myeloid leukemia (AML) cell lines and patient samples compared with normal bone marrow. In addition, RIZ1-deficient mice exhibit a high incidence of diffuse large B-cell lymphoma. In chronic myeloid leukemia (CML), blast transformation has been linked to loss of heterozygosity within chromosomal regions containing RIZ1 [66]. Loss of RIZ1 activity has been reported to impair cellular apoptosis and differentiation while enhancing proliferative capacity, which may contribute to the expansion of bone marrow blast cells and thereby promote CML progression [67].

In contrast, PRDM5, which is generally considered a tumor suppressor in many solid tumors, appears to exert a distinct role in hematologic malignancies. Elevated PRDM5 expression has been independently associated with poorer overall survival in patients with AML. Functional studies indicate that PRDM5 overexpression promotes cell proliferation, colony formation, and migration in vitro, and enhances tumorigenicity in xenograft models in vivo. Mechanistically, PRDM5 has been reported to act as an oncogenic driver in AML through activation of the JNK (c-Jun N-terminal kinase) signaling pathway, suggesting that PRDM5 may represent a potential therapeutic target in this disease [35].

PRDM11 has also been implicated in the pathogenesis of hematologic malignancies, particularly diffuse large B-cell lymphoma (DLBCL). Loss of PRDM11 has been reported to cooperate with MYC overexpression to promote DLBCL progression, and PRDM11-deficient DLBCLs are associated with poorer overall survival and are predominantly classified within the non-germinal center B-cell-like (non-GCB) subtype. Mechanistically, PRDM11 is enriched at transcription start sites of its target genes and regulates the expression of key oncogenic transcription factors, including FOS and JUN. Consistent with a tumor-suppressive role, enforced PRDM11 expression inhibits cell proliferation and induces apoptosis in lymphoma cells [38].

PRDM14 is expressed in embryonic stem cells, primordial germ cells, and a variety of cancers, where it has been shown to confer stem cell-like properties on tumor cells [68]. Functionally, PRDM14 promotes the proliferation of stem cell-like populations while simultaneously sustaining DNA damage, a process that may contribute to genomic instability during tumor development [69]. In hematologic malignancies, PRDM14 has been characterized as a proto-oncogene in T-cell acute lymphoblastic leukemia (T-ALL), where it expands progenitor cell populations and promotes a permissive epigenetic state for oncogenic driver mutations such as NOTCH1, thereby accelerating leukemogenesis [41]. Collectively, these findings suggest that different members of the PRDM family contribute to the initiation and progression of hematologic malignancies through diverse molecular mechanisms.

### 4.2. Autoimmunity and Chronic Inflammation

**Figure 3:**
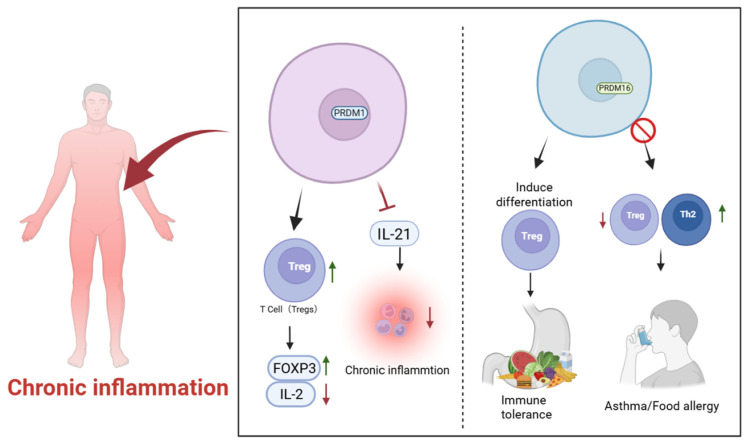
PRDM family members in immune tolerance and chronic inflammation. PRDM1 promotes Treg differentiation and stabilizes FOXP3 expression while suppressing IL-21–mediated inflammatory responses, thereby limiting chronic inflammation. PRDM16 regulates immune tolerance through PRDM16^+^RORγt^+^ tolerogenic dendritic cells that induce peripheral Treg differentiation. Loss of PRDM16 enhances Th2-mediated inflammation and contributes to allergic diseases such as asthma and food allergy.

In the context of autoimmune and chronic inflammatory diseases, PRDM1 (Blimp-1) plays a central role in maintaining immune homeostasis through multiple regulatory mechanisms (Figure 3). On the one hand, it promotes immune tolerance by enhancing the differentiation and function of regulatory T cells (Tregs) and establishing a tolerogenic transcriptional program that limits inappropriate immune activation [27]. On the other hand, PRDM1 restrains excessive inflammatory responses by suppressing the overexpression of pro-inflammatory cytokines, thereby contributing to the control of chronic inflammation [70]. Multi-omics analyses further demonstrate that Blimp-1 can reshape the epigenetic and transcriptional landscape of T cells, stabilizing FOXP3 expression while restricting inflammatory cytokine programs, a process that is critical for maintaining immune tolerance and preventing autoimmune injury such as graft-versus-host disease (GVHD) [71]. In addition, PRDM1 is expressed in thymic epithelial cells (TECs), where it participates in negative selection and the establishment of central immune tolerance; loss of PRDM1 leads to the production of anti-nuclear antibodies and the development of a systemic lupus erythematosus (SLE)-like autoimmune phenotype [72]. Furthermore, Blimp-1 has been shown to suppress IL-21 expression by reducing chromatin accessibility at the IL-21 locus. Given that IL-21 is a key pro-inflammatory cytokine implicated in several autoimmune disorders, including autoimmune diabetes, this regulatory mechanism further highlights the role of PRDM1 in limiting pathogenic immune responses [49].

PRDM16 also plays an important role in immune tolerance and the regulation of inflammatory responses (Figure 3). A distinct subset of PRDM16^+^RORγt^+^ antigen-presenting cells, referred to as tolerogenic dendritic cells (tolDCs), has been identified and shown to induce the differentiation of peripheral regulatory T cells (pTregs), thereby promoting immune tolerance to dietary and commensal microbial antigens. In the absence of PRDM16, pTreg generation is impaired, accompanied by enhanced Th2-mediated and other pro-inflammatory responses. Consequently, PRDM16 deficiency leads to a loss of immune tolerance and exacerbated inflammation in experimental models of asthma and food allergy. These findings indicate that PRDM16 is involved in mechanisms underlying chronic inflammation and immune dysregulation. Notably, this tolDC subset has also been identified in human tissues, suggesting that the underlying regulatory mechanism is evolutionarily conserved and may represent a potential therapeutic target for autoimmune and allergic inflammatory diseases [43].

PRDM3 has also been implicated in the regulation of inflammatory responses. In the context of tumorigenesis, its expression has been reported to correlate with immune cell infiltration and the expression of immune-related markers. In addition, PRDM3 shows positive associations with molecules involved in regulatory T cell (Treg) signaling, including STAT5B and TGF-β1, suggesting that PRDM3 may contribute to the establishment of an immunosuppressive tumor microenvironment rather than promoting classical pro-inflammatory signaling pathways [33]. Although direct evidence linking PRDM3 to canonical autoimmune diseases remains limited, these observations indicate that PRDM3 may participate in the regulation of innate immune-associated inflammatory signaling and could have potential immunopathological relevance.

In addition to the diseases discussed above, PRDM family members also contribute to inflammatory conditions, such as Psoriasis. Dysregulated expression of PRDM2, PRDM3, and PRDM8 in psoriatic lesions suggests their involvement in T cell regulation, keratinocyte proliferation, and trained immunity [37]. Notably, isoform-specific expression patterns, including PR domain-deficient variants, indicate complex and context-dependent functions of PRDM proteins in inflammatory pathogenesis.

### 4.3. Inflammation-Metabolism-Immune Crosstalk in Disease

#### 4.3.1. Osteoporosis

**Figure 4:**
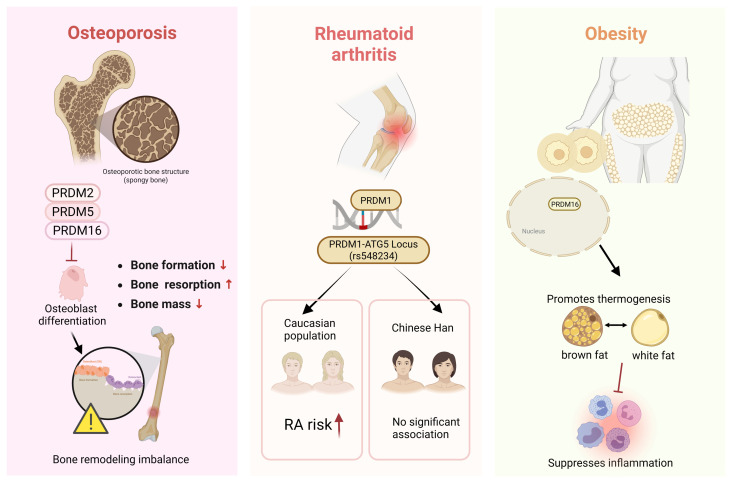
PRDM family members in inflammation–metabolism–immunity crosstalk. PRDM proteins participate in multiple diseases involving metabolic and immune interactions. PRDM2, PRDM5, and PRDM16 regulate osteoblast differentiation and bone remodeling in osteoporosis. PRDM1 genetic variants at the PRDM1–ATG5 locus are associated with rheumatoid arthritis susceptibility in specific populations. PRDM16 regulates thermogenic adipocyte differentiation, enhances energy expenditure, and suppresses inflammation in adipose tissue, thereby influencing obesity and metabolic homeostasis.

Osteoporosis is a systemic skeletal disorder characterized by reduced bone mineral density and deterioration of bone microarchitecture [73]. Traditionally, its pathogenesis has been primarily attributed to an imbalance in bone remodeling, particularly the dysregulated activity between osteoblasts and osteoclasts [74,75]. However, accumulating evidence indicates that bone tissue is closely interconnected with the immune system. The concept of osteoimmunology highlights the complex interactions between immune cells, inflammatory mediators, and bone cells, which collectively play a crucial role in maintaining bone homeostasis as well as in the development of skeletal pathologies [76,77]. In recent years, increasing attention has been directed toward the involvement of PRDM family members in the regulation of bone metabolism and skeletal homeostasis (Figure 4).

Beyond its well-established tumor suppressor function, PRDM2/RIZ1 also plays a role in estrogen-responsive tissues. In particular, RIZ1 acts as a potent coactivator of estrogen receptorα (ERα), thereby influencing bone mineralization. Genome-wide association studies investigating genes linked to osteoporosis have identified PRDM2 as a candidate gene associated with increased osteoporosis risk, suggesting that it may contribute to the regulation of bone mineral density and bone metabolism [78].

PRDM5 is a key regulator of extracellular matrix (ECM) gene transcription in osteoblasts. It is highly expressed in these cells and directly controls the expression of collagen I and other ECM genes by maintaining RNA polymerase II occupancy at their promoters, thereby ensuring proper transcription. Loss of PRDM5 disrupts osteoblastic ECM assembly, delays ossification, and reduces bone mass [79], implicating its potential role in the pathogenesis of osteoporosis.

PRDM3 and PRDM16 can promote bone formation by regulating osteoblast gene expression and modulating the Wnt/β-catenin signaling pathway [80], suggesting that they may have functional significance in conditions of bone metabolic imbalance, such as osteoporosis. Human studies indicate that in hypogonadal men, testosterone therapy upregulates PRDM16, which is associated with enhanced RUNX2 and osteogenic signaling, potentially explaining the molecular basis for increased bone mineral density [81]. In animal models, the Mel1/Prdm16 gene appears to participate in early suppression of Runx2 during osteochondral differentiation, thereby facilitating cartilage formation. PRDM16-deficient mice exhibit skeletal abnormalities, including shortened long bones and impaired cartilage and bone development, highlighting its regulatory role in bone formation and skeletal development [82].

Emerging evidence suggests that additional PRDM family members may also contribute to bone development and metabolic regulation, warranting further investigation in conditions such as osteoporosis. PRDM6 is known to regulate histone methylation and influence neural crest cell (NCC) lineage specification. Although no mechanistic studies have directly demonstrated a role for PRDM6 in osteogenic cells or bone metabolism, genome-wide association and phenotypic GWAS data indicate a trend linking PRDM6 to bone mineral density-related traits [36], suggesting that it may impact skeletal development or metabolic phenotypes. PRDM proteins generally contain a PR/SET domain and can modulate the chromatin state of target genes either autonomously or through interactions with chromatin remodeling complexes, playing critical roles in the differentiation of multiple cell lineages. This epigenetic and lineage-regulatory function shares a common mechanistic basis with the determination of osteoblast and chondrocyte fate, as well as the balance between osteoblast and osteoclast activity [8], Accordingly, less-studied PRDM family members represent potential regulatory candidates in the pathogenesis of osteoporosis and merit detailed investigation in future studies.

#### 4.3.2. Rheumatoid Arthritis

Rheumatoid arthritis (RA) is a chronic systemic autoimmune disease characterized by synovial inflammation, progressive joint destruction, and persistent immune-mediated inflammatory responses [83,84]. Increasing evidence suggests that the pathogenesis of RA involves not only aberrant activation of immune cells but also profound immunometabolic reprogramming. The dynamic interplay between metabolic pathways and immune responses is now recognized as a key driver of disease initiation and progression [85].

Recent genome-wide association studies (GWAS) have identified a significant association between the rs548234 polymorphism located within the PRDM1–ATG5 locus and susceptibility to rheumatoid arthritis (RA) in Caucasian populations, suggesting that PRDM1 may represent a potential genetic risk factor for RA [86]. However, studies conducted in Chinese Han populations have not detected a significant association between this variant and RA susceptibility or clinical phenotypes, either in the overall cohort or after stratification by rheumatoid factor (RF) and anti-cyclic citrullinated peptide (anti-CCP) antibody status [87]. These findings indicate that the relationship between the PRDM1–ATG5 rs548234 variant and RA susceptibility may be population-specific (Figure 4).

Beyond PRDM1, direct genetic or functional evidence linking other members of the PRDM family to RA remains limited. Nevertheless, their established roles in immune regulation, inflammatory signaling, and immune cell function suggest that they may indirectly contribute to the immunopathology of RA. Previous studies have shown that PRDM2/RIZ functions as a transcriptional regulator during T-cell activation and differentiation, exhibiting functional coupling with key signaling pathways such as PI3K and MAPK and participating in the remodeling of immune-related transcriptional programs [28]. Given that the PI3K-AKT and MAPK pathways play central roles in RA by driving aberrant T-cell activation, amplifying inflammatory responses, and promoting pathological remodeling of the synovium [88,89], PRDM2 may influence RA pathogenesis indirectly through modulation of T-cell signaling and transcriptional reprogramming. Therefore, despite the current lack of direct evidence, the potential involvement of PRDM2 and other PRDM family members in RA warrants further systematic investigation.

#### 4.3.3. Obesity

Obesity and its associated metabolic disturbances represent a prominent manifestation of metabolic–immune crosstalk disorders [90], with the regulation of energy homeostasis in adipose tissue constituting a central underlying mechanism [91]. Under obese conditions, adipose tissue functions not merely as an energy storage site but rather as a highly active endocrine and immunoregulatory organ. This functional transition is accompanied by extensive structural and metabolic remodeling, characterized by immune cell infiltration, sustained production of inflammatory mediators, and disruption of metabolic homeostasis [92]. A large body of evidence indicates that both the abundance and phenotypic composition of immune cells within adipose tissue—particularly macrophages and T cells—are markedly altered during obesity. Persistent activation of pro-inflammatory immune responses promotes a state of chronic low-grade inflammation, which in turn interferes with insulin signaling and lipid metabolic processes [93,94,95]. Consequently, the dynamic regulation of adipose tissue energy balance and its immune microenvironment is widely considered a critical mechanism linking obesity with chronic inflammation and metabolic dysregulation.

Among PRDM family members, PRDM16 has emerged as a particularly prominent regulator in this context (Figure 4). Acting as a key transcriptional regulator, PRDM16 plays a central role in the differentiation and functional maintenance of brown adipose tissue (BAT) and beige adipocytes. Its overexpression markedly increases energy expenditure, limits high-fat diet-induced weight gain, and improves glucose tolerance, whereas loss of PRDM16 leads to reduced expression of thermogenic genes and promotes the development of an obese phenotype [44]. Beyond its direct activation of thermogenic gene programs, PRDM16 also contributes to metabolic regulation through suppression of inflammatory signaling pathways. In adipocytes, PRDM16 has been shown to inhibit type I interferon (IFN) signaling, thereby preventing IRF1-mediated activation of pro-inflammatory target genes. Through this mechanism, PRDM16 preserves mitochondrial function and thermogenic capacity, highlighting its role as a molecular link between metabolic regulation and inflammatory signaling in adipose tissue [45]. PRDM16 also cooperates with several transcriptional cofactors, including PPARγ, PGC-1α [96], and NFIA [97], to regulate the gene program of thermogenic adipocytes, while the PRDM16-GTF2IRD1/EHMT1 transcriptional complex represses obesity-associated adipose tissue fibrosis [98,99], thereby improving systemic glucose homeostasis and metabolic phenotypes.

Clinical observations together with cellular evidence further support an important role for PRDM16 in obesity and metabolic regulation. Studies have shown that PRDM16 expression is significantly reduced in adipose tissue of individuals with obesity and is associated with decreased insulin sensitivity. Moreover, the expression of PRDM16 can be modulated by insulin-sensitizing agents, such as metformin and rosiglitazone [100]. These findings suggest that, beyond its role in adipose energy metabolism, PRDM16 may represent a potential therapeutic target for the treatment of obesity and metabolic syndrome. By promoting thermogenic programming, PRDM16 can improve adipose tissue metabolic status and attenuate local inflammation, which may in turn influence systemic inflammatory responses.

Accumulating evidence suggests that, in addition to PRDM16, other members of the PRDM family may also participate in the regulation of obesity-related energy metabolism. For example, PRDM4 has been reported to regulate energy expenditure and the expression of beige adipocyte-associated genes in adipose tissue, and its deficiency exacerbates high-fat diet-induced obesity, indicating a positive role for PRDM4 in metabolic regulation [34]. Although direct high-impact evidence linking PRDM3 to obesity remains limited, mechanistic studies indicate that both PRDM3 and PRDM16 can interact with the transcriptional coactivator MED1 and contribute to the regulation of brown adipose-specific gene expression [101]. Under conditions of PRDM16 deficiency, PRDM3 is able to partially compensate for the regulation of thermogenic gene programs, suggesting that PRDM3 may indirectly participate in obesity-related metabolic regulation through similar transcriptional mechanisms and potentially influence immunometabolic balance. In addition, genome-wide association studies (GWAS) have identified multiple genetic loci associated with obesity, including PRDM6 [102], providing new insights into the genetic determinants and complex regulatory networks underlying obesity.

## 5. Conclusions 

The PRDM family comprises a group of transcriptional and epigenetic regulators characterized by the presence of a PR/SET domain. These proteins play essential roles in the immune system by governing cell lineage determination, functional differentiation, and the maintenance of immune homeostasis. Through subtype-specific regulatory mechanisms, PRDM proteins modulate chromatin architecture and transcriptional programs that control the development and function of both innate and adaptive immune cells, while also contributing to the regulation of inflammatory responses and immune tolerance. Emerging evidence further indicates that PRDM family members are involved in the pathogenesis of hematological malignancies, autoimmune disorders, and immunometabolic diseases, including obesity and osteoporosis. A comprehensive understanding of these findings provides an integrated view of the biological significance of PRDM proteins in immune regulation and offers a conceptual framework for exploring their roles in disease.

Despite the growing body of evidence supporting the roles of PRDM family proteins in immune regulation, several limitations should be acknowledged. First, the current understanding of PRDM function is highly uneven across family members, with PRDM1/Blimp-1 being extensively studied, while many other PRDM proteins remain poorly characterized. Second, findings from different studies are sometimes inconsistent or even contradictory, likely due to variations in experimental models, cell types, and disease contexts. For example, certain PRDM proteins have been reported to exert both pro-inflammatory and anti-inflammatory effects depending on the cellular environment, highlighting the context-dependent nature of their regulatory functions. Moreover, most available studies are based on in vitro systems or animal models, and there is still a lack of well-controlled human studies to validate these findings. In addition, the potential compensatory or redundant functions among PRDM family members have not been fully explored. Future investigations should therefore aim to clarify the cell type-specific functions of PRDM proteins and define their roles within broader transcriptional and epigenetic regulatory networks. Advances in high-resolution approaches, including single-cell transcriptomics, epigenomic profiling, and functional genetic models, will provide valuable tools to dissect PRDM-dependent regulatory circuits in immune cells. A deeper understanding of these mechanisms may ultimately facilitate the identification of new molecular targets for therapeutic intervention in hematological, autoimmune, and immunometabolic diseases.

Importantly, current studies on PRDM family proteins in infection-related immunity remain very limited. The roles of PRDM members in host–pathogen interactions, innate immune activation, and antimicrobial responses are still largely unexplored. Further investigations are required to elucidate their potential regulatory mechanisms in infection-associated immune processes.

Therefore, future studies should focus on systematically characterizing less-studied PRDM proteins, clarifying their context-specific roles in different immune cell subsets, and integrating multi-omics approaches to better understand their regulatory networks. Furthermore, well-designed in vivo and clinical studies are needed to evaluate their translational potential in immune-related diseases.

## Figures and Tables

**Table 1:** Structural features and immune-related functions of PRDM family members.

PRDM Member	Structural Features	Immune-Related Functions	References
PRDM1 (BLIMP-1)	PR domain + C2H2 zinc fingers	B cell terminal differentiation; T cell functional regulation; NK cell homeostasis; immune tolerance	[23,24,25,26,27]
PRDM2 (RIZ1/RIZ2)	PR domain-containing isoform (RIZ1) and PR-lacking isoform (RIZ2)	B cell differentiation; T cell activation regulation	[28,29,30]
PRDM3 (MECOM)	PR domain + multiple zinc fingers; interacts with chromatin remodeling complexes	Regulates hematopoietic stem cell maintenance and lineage commitment; associated with immune cell differentiation and immune-related tumor microenvironment regulation	[20,31,32,33]
PRDM4	PR domain + zinc fingers	Potential role in immunometabolism and energy balance; may indirectly influence immune responses through metabolic regulation	[34]
PRDM5	PR domain-containing transcription factor regulating extracellular matrix gene expression	Involved in tumor-associated immune regulation; promotes proliferation and migration	[13,35]
PRDM6	PR domain-containing protein with histone methylation-related activity	Limited direct evidence	[36]
PRDM7	PR domain + zinc fingers; closely related to PRDM9	Currently unclear; no direct evidence in immune regulation	[10]
PRDM8	PR domain + zinc fingers; transcriptional regulator	Potential role in inflammatory diseases	[37]
PRDM9	PR domain + zinc fingers	No direct role in immune regulation reported	[10]
PRDM10	PR domain-containing transcription factor	Limited evidence; may participate in general transcriptional regulation	[10]
PRDM11	PR domain + zinc fingers	Regulates T cell differentiation and lymphomagenesis; tumor suppressor in B-cell lymphoma	[38]
PRDM12	PR domain-containing transcription factor	Primarily involved in neural development; no clear immune-related function	[10]
PRDM13	PR domain + zinc fingers	Limited evidence; mainly involved in neural lineage specification	[10]
PRDM14	PR domain-containing transcriptional regulator	Regulates lymphoid progenitor expansion	[39,40,41]
PRDM15	PR domain-containing transcription factor	Limited evidence; may regulate early developmental and metabolic pathways	[10]
PRDM16	PR domain + zinc fingers	Regulates hematopoietic stem cells, immune tolerance	[42,43,44,45]
FOG1	Zinc finger protein; lacks PR domain	Regulates hematopoiesis, particularly erythroid and megakaryocyte differentiation	[10]
FOG2	Zinc finger protein; lacks PR domain	Limited direct immune role; involved in developmental processes	[10]

## Data Availability

The authors have reviewed and edited the output and take full responsibility for the content of this publication.

## Abbreviations

AML	acute myeloid leukemia
BAT	brown adipose tissue
BLIMP-1	B lymphocyte-induced maturation protein 1
CML	chronic myeloid leukemia
DCs	dendritic cells
DLBCL	diffuse large B-cell lymphoma
ECM	extracellular matrix
GVHD	graft-versus-host disease
GWAS	genome-wide association studies
HSCs	hematopoietic stem cells
MAMPs	microbe-associated molecular patterns
NK cells	natural killer cells
PRDM	PRDI-BF1 and RIZ homology domain-containing protein
PRRs	pattern recognition receptors
RA	rheumatoid arthritis
SLE	systemic lupus erythematosus
T-ALL	T-cell acute lymphoblastic leukemia
TECs	thymic epithelial cells
Th cells	T helper cells
tolDCs	tolerogenic dendritic cells
Tregs	regulatory T cells
pTregs	peripheral regulatory T cells
